# Effective Control of Neuropathic Pain With Amitriptyline in Neuromyelitis Optica Spectrum Disorder: A Case Report

**DOI:** 10.7759/cureus.87639

**Published:** 2025-07-09

**Authors:** Koji Hayashi, Koichi Kimura, Yuka Nakaya, Asuka Suzuki, Mamiko Sato, Naoko Takaku, Toyoaki Miura, Kosuke Misaki, Yasutaka Kobayashi

**Affiliations:** 1 Department of Rehabilitation Medicine, Fukui General Hospital, Fukui, JPN; 2 Graduate School of Health Science, Fukui Health Science University, Fukui, JPN; 3 Department of Neurology, University of Fukui, Fukui, JPN; 4 Department of Orthopedics, Fukui General Hospital, Fukui, JPN

**Keywords:** amitriptyline, antidepressant drug, neuromyelitis optica spectrum disorder (nmosd), neuropathic pain, pain management

## Abstract

We describe a neuromyelitis optica spectrum disorder (NMOSD) case with neuropathic pain (NP) that was successfully controlled by amitriptyline. A 48-year-old woman presented with progressive left lower limb pain, gait disturbance, and sensory deficits, alongside a history of bilateral posterior thigh numbness. Neurological examination revealed motor weakness, hyperreflexia, positive Babinski signs, sensory impairments, and bladder and rectal dysfunction. Laboratory tests showed elevated serum anti-aquaporin-4 (anti-AQP4) antibody levels, and MRI demonstrated longitudinally extensive spinal cord lesions consistent with NMOSD. The patient was treated with methylprednisolone pulse therapy, followed by maintenance immunosuppressive therapy with inebilizumab and prednisolone, resulting in neurological improvement. However, around 15 weeks after onset, the patient experienced worsening burning sensations and numbness from the umbilicus to the toes, significantly impairing sleep due to NP (Numeric Rating Scale, or NRS: 7-8). Despite stable neurological findings and no new MRI abnormalities, she was diagnosed with NP related to NMOSD. Commencing amitriptyline at 5 mg/day led to rapid symptom relief by the next day, effectively controlling her NP. The pain remained well-managed with continued amitriptyline, allowing a gradual reduction in steroid dosage without symptom recurrence.

This case highlights the potential of amitriptyline as an effective treatment for NP in NMOSD, a common and often resistant symptom in this condition. Given the complex mechanisms underlying NMOSD-related pain, individualized management strategies that include pharmacological agents like amitriptyline are crucial for improving patient quality of life (QOL), and further research is needed to optimize these approaches.

## Introduction

Neuromyelitis optica spectrum disorder (NMOSD), also known as Devic disease, is a severe autoimmune inflammatory disorder of the central nervous system (CNS) [1-3]. It occurs in individuals of all ethnicities [1]. NMOSD primarily targets the optic nerves and spinal cord [2,4-7]. Although traditionally focused on these regions, numerous studies have subsequently demonstrated that NMOSD can also involve the brain, brainstem, and other CNS regions [1,4-6,8]. The disease is characterized by recurrent episodes or attacks, interspersed with periods of remission [2,4,6,7]. Recovery after attacks is often incomplete, leading to cumulative neurological disabilities and persistent, severe symptoms [7].

Among many cases of NMOSD, the presence of pathogenic immunoglobulin G (IgG) antibodies against the astrocytic water channel aquaporin-4 (AQP4) is frequently observed [1,6,9]. AQP4 is predominantly localized to astrocytic processes [1]. AQP4-IgG antibodies are considered highly specific biomarkers for NMOSD and play a key role in initiating and propagating the characteristic astrocyte-destructive lesions [1,2]. Although most patients with NMOSD are AQP4-IgG seropositive, some are seronegative for this antibody [1,7,8]. AQP4-IgG seropositivity is widely used for diagnosis [2,3,9]. Major clinical presentations typically include optic neuritis (ON) and longitudinally extensive transverse myelitis (LETM); however, symptoms such as pain or painful tonic spasms (PTS) can also develop, adversely affecting quality of life (QOL) [1,2,6-9].

Amitriptyline is a tricyclic antidepressant that is widely used and often recommended as a first-line treatment for chronic neuropathic pain (NP) [10]. Although decades of clinical experience suggest successful outcomes in many patients, high-quality, unbiased evidence supporting its significant pain-relieving effect is considered limited [10]. Nevertheless, many clinicians prescribe this medication for NP management. It should be noted, however, that amitriptyline does not work for all NP patients [10].

In this report, we present a compelling case of AQP4-IgG seropositive NMOSD characterized by numbness, dysesthesia, and pain, which was effectively managed with amitriptyline - highlighting its potential as a valuable therapeutic option for NP in NMOSD patients.

## Case presentation

A 48-year-old woman, with a history of bilateral posterior thigh numbness attributed to lumbar spondylosis, presented to our hospital with a three-day history of progressively worsening left lower limb pain and gait disturbance. She had a previous episode at age 47, characterized by intractable hiccups lasting approximately one week; investigations at another facility, including tests for connective tissue disease markers, were inconclusive. Neurological examination revealed normal vision and muscle strength of Medical Research Council (MRC) Grade 5 in the upper limbs. However, muscle strength in the right lower limb was assessed at MRC Grade 4, while the left lower limb was at MRC Grade 1. Both lower extremities exhibited hyperreflexia and positive Babinski signs. Additionally, the patient showed superficial sensory impairment distal to the umbilicus on the left side and distal to the inguinal region on the right. She also exhibited impaired proprioception in the left toes, and bladder and rectal dysfunction.

**Figure 1:**
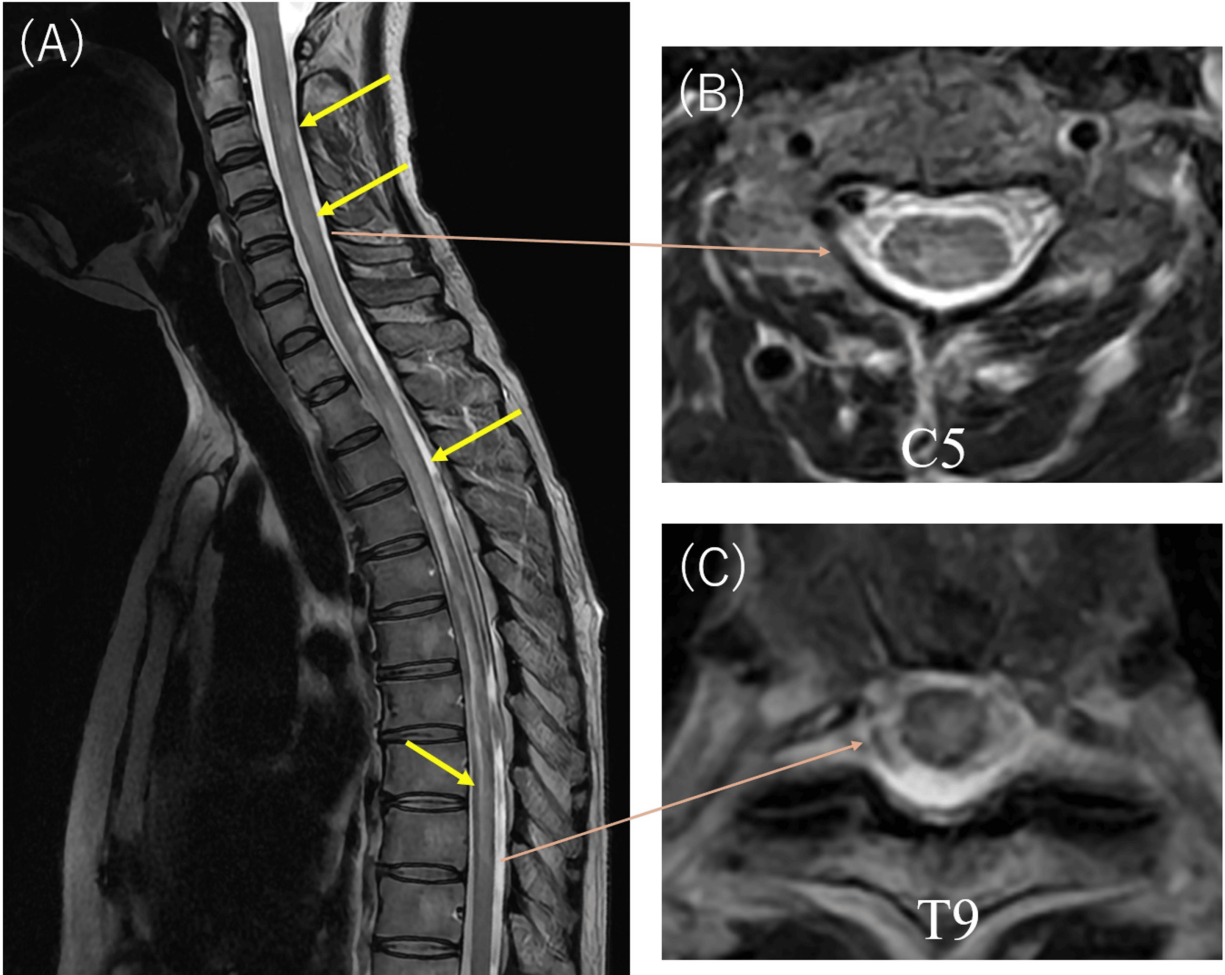
Result of spinal MRI (A) Spinal MRI using T2-weighted, fat-suppressed fast spin echo sequences demonstrates long cord lesions extending from the cervical to the thoracic spinal cord (yellow arrows). (B, C) Axial sections through the cervical and thoracic spinal cord reveal transverse abnormal signals within the cord (C5 and T9 levels shown, orange arrows).

Laboratory tests indicated elevated white blood cell count, creatine phosphokinase (CPK), and C-reactive protein (CRP), along with slight hypoalbuminemia and hyponatremia. Serum anti-AQP4 antibody levels were markedly elevated (≥40.0 U/mL), whereas human T-cell lymphotropic virus type 1 (HTLV-1) antibodies were negative (Table 1). Cerebrospinal fluid tests revealed albuminocytologic dissociation (cell count: 4/µL; protein: 66.7 mg/dL) and elevated myelin basic protein (383.8 µg/L; reference: <1.5 µg/L). Whole-spinal MRI revealed a longitudinally extensive T2 hyperintense lesion extending from the cervical to thoracic spinal cord (Figure 1). No notable abnormalities were observed on brain MRI. Based on these findings, a diagnosis of NMOSD was made.

**Table 1:** Results of blood tests on admission

Inspection items	Result	Reference range
White blood cell count	12300 /μL	3300-8600
Red blood cell count	436 × 10^4^ /μL	386-492 × 10^4^
Hemoglobin	13.6 g/dL	11.6-14.5
Blood platelet	25.5 × 10^4^ /μL	15.8-34.8
Total protein	7.5 g/dL	6.6-8.1
Albumin	3.9 g/dL	4.1-5.1
Glucose	103 mg/dL	73-109
Blood urea nitrogen	14.8 mg/dL	8.0-20.0
Creatinine	0.57 mg/dL	0.46-0.79
Total bilirubin	0.6 mg/dL	0.4-1.2
Aspartate aminotransferase	23 U/L	13-30
Alanine aminotransferase	15 U/L	7-30
Alkaline phosphatase	72 U/L	38-113
Lactate dehydrogenase	200 U/L	124-222
γ-glutamyltransferase	17 U/L	13-64
Creatine phosphokinase	302 U/L	41-153
Choline esterase	387 U/L	240-486
Amylase	112 U/L	44-132
Sodium	137 mmol/L	138-145
Potassium	4.0 mmol/L	3.6-4.8
Chlorine	101 mmol/L	101-108
C-reactive protein	2.43 mg/dL	0.00-0.14
Anti-aquaporin-4 antibody	>40.0 U/L	0.0-2.9
Human T-lymphotropic virus 1 antibody	-	-

Treatment was initiated with two courses of methylprednisolone pulse therapy (1000 mg/day for three days), followed by maintenance therapy with inebilizumab and oral prednisolone at 1 mg/kg/day. The patient responded well to steroid therapy, and her neurological deficits improved, including a reduction in lower limb pain and an increase in activities of daily living (ADL). Neurological examination at 14 weeks after onset demonstrated muscle strength of MRC Grade 4-5 in the right lower limb and Grade 3-4 in the left lower limb. The patient was able to ambulate independently with the assistance of a T-shaped cane and exhibited numbness in the dorsum of both feet.

**Figure 2:**
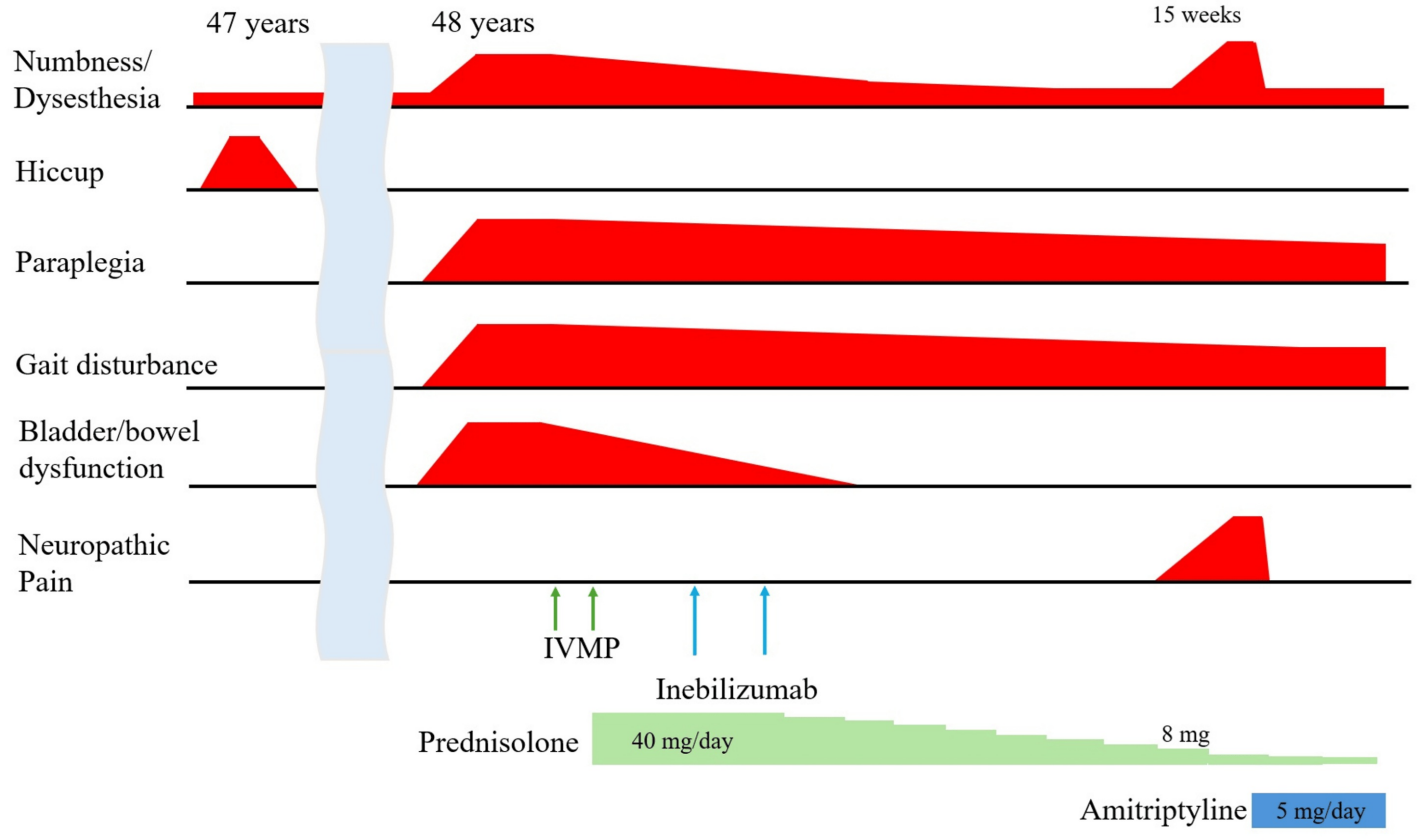
A diagram showing the patient's symptoms and treatment progress The patient had experienced numbness in both lower limbs due to lumbar spondylosis for some time. She previously experienced an unexplained hiccup at age 47. At age 48, she developed spinal cord symptoms, including numbness, abnormal sensations, paresis, gait disturbance, and bladder and rectal dysfunction. After two rounds of intravenous methylprednisolone pulse therapy and treatment with inebilizumab, these symptoms improved. Around 15 weeks after onset, her numbness and pain in both lower limbs worsened. Upon further examination, the pain was diagnosed as neuropathic pain. Since it significantly impaired her quality of life, treatment with amitriptyline 5 mg/day was initiated, resulting in a remarkable improvement.

Around the 15th week after onset, the dose of prednisolone was reduced to 8 mg/day. She experienced worsening of a burning sensation and numbness from the umbilicus to the toes, which interfered with her sleep due to pain (Numeric Rating Scale, or NRS: 7-8). Objective assessments of her muscle strength and gait ability showed no significant changes. Thoracic MRI revealed no changes compared to the MRI on admission. CSF tests showed no pleocytosis (cell count: 1/µL) and mildly elevated protein (45.5 mg/dL). Based on these findings, she was diagnosed with NP associated with NMOSD, rather than a recurrence of the disorder. She was prescribed amitriptyline 5 mg/day. Following the initiation of this treatment, the burning sensation and numbness subsided by the next day, returning to her previous baseline. She continued to reduce her prednisolone dose to 5 mg, but there was no worsening of the numbness. The symptoms have been well controlled with amitriptyline 5 mg/day. A timeline table is shown in Table 2, and a schematic showing the progression of these symptoms is shown in Figure 2.

**Table 2:** Timeline of key events NMOSD: Neuromyelitis Optica Spectrum Disorder; NRS: Numeric Rating Scale

Time	Event
Week 0	NMOSD diagnosis, methylprednisolone initiated
Week 14	Improved neurological function, ambulating with a cane
Week 15	Prednisolone tapered to 8 mg/day, pain exacerbation (NRS 7-8)
Week 15 (post amitriptyline)	Amitriptyline 5 mg/day initiated, burning sensation and numbness subsided to baseline the next day
Week 16	Prednisolone tapered to 5 mg, symptoms well controlled under amitriptyline 5 mg/day

## Discussion

To our knowledge, this case represents the first report documenting the efficacy of amitriptyline for NP associated with NMOSD. The patient was positive for anti-AQP4 antibodies and had a long spinal cord lesion, making the diagnosis of NMOSD straightforward. Her history included hiccups one year prior to admission, which may have been related to the manifestation in the area postrema of the dorsal medulla in NMOSD [11]. She had received standard treatments, including methylprednisolone pulse therapy, oral prednisolone, and inebilizumab. Oral prednisolone was initiated at 40 mg/day (1 mg/kg/day) and was gradually tapered after inebilizumab administration, reducing to 8 mg/day. During this period, she experienced increased pain and burning sensations in both lower limbs. MRI and CSF findings did not show any exacerbation, and there were no neurological changes other than increased numbness, suggesting that the symptoms were more consistent with heightened NP rather than a relapse of NMOSD. Notably, her symptoms improved dramatically with amitriptyline, and she was subsequently followed up without any worsening of paralysis or deterioration of bladder or rectal function. We chose amitriptyline because the European Federation of Neurological Societies (EFNS) guidelines for level A drug therapy of NP state that tricyclic antidepressants, including amitriptyline, are effective for diabetic NP or post-herpetic neuralgia [12].

Pain is a common and often severe symptom in NMOSD [9], with more than 80% of patients experiencing chronic pain [9]. Pain in NMOSD is typically intense and intractable, markedly reducing patients’ QOL [9]. Many patients report pain as their most disabling symptom [4], and some studies suggest that pain can impair QOL as much as disability and motor weakness [8]. Importantly, pain in NMOSD often resists standard treatments, indicating that the underlying mechanisms may differ from those involved in other treatable pain conditions [1].

NP appears to be the most prevalent form of pain in NMOSD, reported in 41.6% to 90.9% of patients [5]. The features of NP in NMOSD include sensations such as burning, tingling, prickling, electric shock-like, and stabbing pains [5,6]. Another notable pain manifestation in NMOSD is PTS, characterized by paroxysmal episodes of increased muscle tone, abnormal postures, and intense pain, occurring in approximately 22.61% to 26.66% of NMOSD cases [2].

Regarding the treatment of pain related to NMOSD, pain in NMOSD is often refractory to treatment [1,5,7]. Standard-of-care pain medications include antiepileptics, opioids, and nonsteroidal anti-inflammatory agents [1]. Oxcarbazepine is recommended as a first-line treatment for PTS in NMOSD [2]. A report suggests monoamine reuptake inhibitors may be beneficial in NMO [1]. However, the effectiveness of these standard treatments for NP in NMOSD is often unsatisfactory [5]. Immunosuppressive therapies are beneficial not only in preventing disease recurrence in NMOSD but also in alleviating pain symptoms [6].

Worsening pain and numbness during the course of NMOSD treatment may result from non-inflammatory pathologies unrelated to a possible relapse. The severity of pain has been reported to be greater in patients who have experienced a higher number of relapses [7]. New-onset pain, including NP, headache, and ON-related eye pain, frequently occurs during the acute phase of NMOSD attacks and often indicates disease recurrence [6]. Repeated relapses can contribute to worsening pain over time [7]. Conversely, patients may experience more than one type of pain syndrome, and the predominant pain syndrome can change as the disease progresses, even in the absence of new inflammatory activity [8].

In our case, although the pain worsened during the steroid tapering period - raising concerns about possible recurrence - there was no deterioration of other symptoms, and no signs indicative of relapse were observed even after amitriptyline treatment. Therefore, the pain was considered to be non-recurrent (non-inflammatory) NP. While there are very few reports on the efficacy of antidepressants for NMOSD-related NP, amitriptyline may represent a promising option for pain management, similar to its established use in NP of other causes. Its potential effectiveness in NMOSD-related NP is likely related to its mechanism of action as a tricyclic antidepressant, which can enhance descending inhibitory pathways and modulate pain transmission [1]. Specifically, its dual action on serotonin and noradrenaline reuptake may help compensate for impaired endogenous pain control systems, often compromised in NMOSD due to astrocyte dysfunction and neuroinflammatory processes [13]. Although direct clinical data for NMOSD are limited, the proven efficacy of amitriptyline in treating various forms of NP supports its potential utility in this context [1], highlighting the need for further investigation. Further research is necessary to elucidate the effects of amitriptyline on NP associated with NMOSD.

## Conclusions

This case suggests that amitriptyline may be effective in managing non-relapse-related NP associated with NMOSD, highlighting its potential as part of a tailored approach to refractory pain in this context. NMOSD-related pain is common and often resistant to standard therapies, emphasizing the need for further investigation into alternative treatment options. Given the substantial impact of pain on QOL, clinicians should consider individualized pain management strategies - potentially including tricyclic antidepressants such as amitriptyline - alongside immunosuppressive therapies. Additional research is needed to better understand pain mechanisms in NMOSD and to establish optimal management protocols.
